# SG-WAS: A New Wireless Autonomous Night Sky Brightness Sensor

**DOI:** 10.3390/s21165590

**Published:** 2021-08-19

**Authors:** Miguel R. Alarcon, Marta Puig-Subirà, Miquel Serra-Ricart, Samuel Lemes-Perera, Manuel Mallorquín, César López

**Affiliations:** 1 Instituto de Astrofísica de Canarias, C/Vía Láctea s/n, E-38205 La Laguna, Canarias, Spain; marta.puig@iac.es (M.P.-S.); mserra@iac.es (M.S.-R.); samuel.lemes@iac.es (S.L.-P.); manuel.mallorquin.diaz@iac.es (M.M.); 2 Departamento de Astrofísica, Universidad de La Laguna (ULL), E-38206 La Laguna, Canarias, Spain; 3 Sieltec Canarias S.L., C/Hábitat No. 2, Portal D, Of. 3, E-38204 La Laguna, Canarias, Spain; cesar.lopez@sieltec.es; 4 Departamento de Ingeniería Agraria, Naútica, Civil y Marítima, Universidad de La Laguna (ULL), E-38200 La Laguna, Canarias, Spain

**Keywords:** light pollution, night sky brightness, artificial lighting, photometer, skyglow, detection networks

## Abstract

The main features of SG-WAS (SkyGlow Wireless Autonomous Sensor), a low-cost device for measuring Night Sky Brightness (NSB), are presented. SG-WAS is based on the TSL237 sensor –like the Unihedron Sky Quality Meter (SQM) or the STARS4ALL Telescope Encoder and Sky Sensor (TESS)–, with wireless communication (LoRa, WiFi, or LTE-M) and solar-powered rechargeable batteries. Field tests have been performed on its autonomy, proving that it can go up to 20 days without direct solar irradiance and remain hibernating after that for at least 4 months, returning to operation once re-illuminated. A new approach to the acquisition of average NSB measurements and their instrumental uncertainty (of the order of thousandths of a magnitude) is presented. In addition, the results of a new Sky Integrating Sphere (SIS) method have shown the possibility of performing mass device calibration with uncertainties below 0.02 mag/arcsec${}^{2}$. SG-WAS is the first fully autonomous and wireless low-cost NSB sensor to be used as an independent or networked device in remote locations without any additional infrastructure.

## 1. Introduction

The increasing use of the artificial light at night (ALAN) has a dangerous impact on natural ecosystems. It can be so faint that humans cannot see it, but it has been shown that it could still threaten 30% of vertebrates and 60% of invertebrates that are nocturnal and very sensitive to light [1,2,3,4]. Eighty percent of the world’s population lives in places where there is artificial light pollution, and about one-third of them cannot see the Milky Way. There are very few places left on the planet where natural darkness can be appreciated, observed, and measured [5]. The environmental effects of ALAN are not restricted to the direct nocturnal ecosystem impact; there is also some evidence of the role of outdoor lighting in the nocturnal dynamics of chemical pollutants over cities [6].

The emissions of ALAN from cities can be monitored using instruments on Earth-orbiting satellites [7,8]. However, evaluating the effects of these emissions on night sky brightness (NSB) in very dark places (normally natural protected areas or astronomical observatories) requires extensive ground-based observations carried out by photometer networks [9,10]. These networks are also necessary for testing light pollution models that predict NSB as a function of the location and distribution of artificial sources and the scattering of light in the atmosphere [11].

The Unihedron Sky Quality Meter (hereafter SQM), a low-cost and pocket-size NSB photometer, has given the general public the possibility of quantifying the NSB at any place and time [12]. It is a silicon photodiode with a TSL237 light-to-frequency converter and a Hoya CM-500 filter that limits its effective bandpass to 400–650 nm. Based on the same TSL237 photodiode SQM detector, the European funded project STARS4ALL (www.stars4all.eu, accessed on 28 June 2021) developed a new Telescope Encoder and Sky Sensor (TESS [13]), with a more extended spectral response in the red (400–800 nm) to include the emission lines of High Pressure Sodium (HPS) lamps.

Irradiance-to-frequency converter sensor technologies used in SQM and TESS have significantly expanded the range of inexpensive and easy-to-use NSB photometers, without sacrificing the precision and detail of professional instruments [14]. However, although the cost of photometers has dropped, their deployment in remote locations (e.g., protected natural areas) remains a major drawback for many projects. SG-WAS, a new, low-cost NSB photometer based on the TSL237 sensor, is presented in this article, which is organized as follows. In Section 2, a description of the SG-WAS photometer is given, together with a detailed explanation of WAS technology and field tests. The new Sky Integrating Sphere (SIS) method and its results are presented in Section 3, along with a brief discussion of the implications of the results and uncertainties. Finally, the main conclusions are provided in Section 4.

## 2. SG-WAS Photometer

The SkyGlow Wireless Autonomous Sensor (SG-WAS) is a new NSB photometer partially funded by the EU project EELabs, and designed and manufactured by the Canarian R&D company Sieltec Canarias S.L., under the scientific coordination of the Instituto de Astrofísica de Canarias (IAC, Tenerife, Spain). SG-WAS is basically composed of the following.

1. A TSL237 irradiance-to-frequency converter sensor.
2. An optical dichroic that determines the spectral response of the device.
3. A concentrator optic that focuses the light and determines the effective field of view (FOV).
4. An infrared (IR) sensor that measures the sky temperature.
5. Two microcontrollers that convert the frequencies measured into an average magnitude and uncertainty, store and send the information, and control the power-saving strategy.
6. A communication unit (LoRa, WiFi, LTE-M) customizable to the place where it is installed.

**Figure 1:**
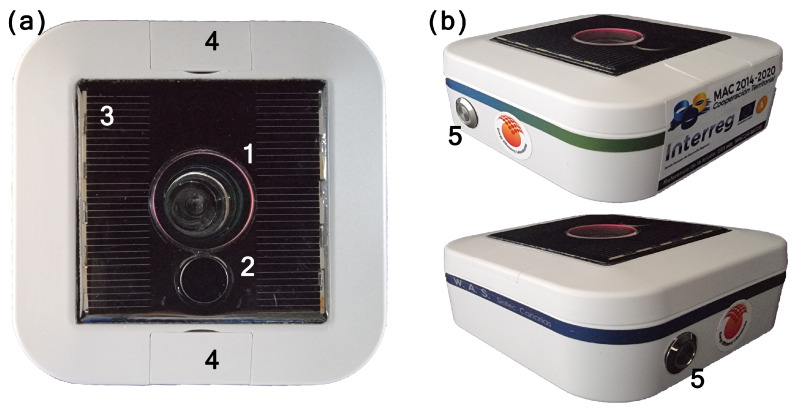
SG-WAS photometer viewed from above (**a**) and two sides (**b**). Its dimensions are 10 cm × 10 cm × 4 cm and it weighs about 100 g. The main elements are sky brightness optics (1), infrared thermometer (2), solar panel (3), fixing screws (4), and power button (5).

The photometer’s dimensions are similar to those of other photometers of its class (10 cm × 10 cm × 4 cm) and its weight is approximately 100 g. It is designed to be placed horizontally on a flat surface (optionally provided by the manufacturer), so its 20 mm-diameter window and the IR sky temperature sensor face the zenith (see Figure 1). On one side of its casing, there is a color-coded manual switch-off button that may be enabled in configuration mode. A photovoltaic cell on the upper part allows it to be recharged within a few hours with direct sunlight (see Section 2.2). The SG-WAS is the first fully autonomous and wireless NSB photometer. It is waterproof and resistant to adverse weather conditions.

### 2.1. Wireless

**Figure 2:**
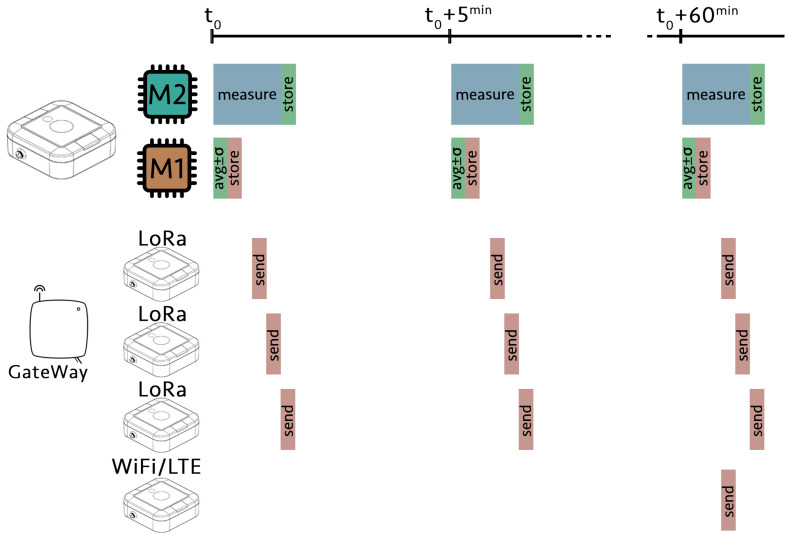
Measurement and communication diagram of the SG-WAS photometer. Each device has two microcontrollers: M2 takes ten NSB measurements and stores them temporarily; M1 calculates the average and uncertainty of the data set taken in the previous slot, and stores them until they are transmitted to the central hub. This process is repeated every 5 min. In the LoRa version, the GateWay assigns a 6 s slot every 5 min to each photometer to send its measurements and synchronize its timestamp for the next cycle. The GateWay sends the measurements of all the devices connected to it to the server. In the WiFi and LTE-M version, the measurements are directly sent to the server once every hour and M1 is continuously synchronized with the network. During the rest of the time, both microcontrollers remain in a deep sleep state, so the battery consumption is extremely low.

In recent decades, there has been a growing interest in installing sensor networks to monitor different variables related to environmental protection [15,16], ecology [17,18], and volcanology [19], among others, in remote or difficult-to-access locations with no infrastructure. These wireless sensor networks (WSN) are organized as a series of dispersed nodes with sensing capabilities that collect and send data to a centralized hub. All SG-WAS photometers are independent NSB sensors conceived to be used as one of these nodes. The measurement and communication process is shown in Figure 2. There are two microcontrollers incorporated:

- M2 is connected to the sensor, takes measurements, and stores them temporarily.
- M1 handles timing, storage, data processing, and communication. It is connected to M2 and the central hub (GateWay in LoRa version or server in WiFi/LTE-M version).

To reduce power consumption as much as possible, two independent processes are carried out:

1. Every 5 min, M1 calculates the average and instrumental uncertainty of the ten measurements taken by M2 in the previous slot, stores them, and commands M2 to take a new set of measures.
2. In the corresponding slot, depending on the device version, M1 encrypts, and sends the measurements stored in its memory to the central hub. These include the average and standard deviation of the ten continuous NSB measurements, ambient and sky temperature, battery charge, and communication signal strength.

During the rest of the time, both microcontrollers remain in a deep sleep state, so the battery consumption is extremely low. Different versions of SG-WAS depend on the communication protocol (WiFi, LoRa, or LTE-M), the connection to the central hub and how the sending slots are assigned.

#### 2.1.1. LoRa Version

LoRa [20] is a patented wireless communication technology commercialized by Semtech Corporation since 2012. Signals are modulated in sub-GHz license-free Industrial, Scientific, and Medical (ISM) radio bands, making it affordable, deployable worldwide, and interoperable. With its ability to reach long distances of up to 15 km in rural areas, provide data rates in the range of kilobits per second, and more inexpensive base stations (GateWay), LoRa is a very attractive technology for WSN communication in remote locations.

Although there are some ALOHA-based communication protocols with random access channels—LoRaWAN [21] is one of most widely known—that support networks with thousands of sensors connected to the same GateWay, networks with densities of less than one photometer per square kilometer are not expected [9]. Therefore, each GateWay is limited to several tens of photometers. In addition, random access channels require data re-sending or active listening strategies to ensure the reception of all the packets, which is a power-consuming process. Consequently, a deterministic communication protocol of Time Division Multiple Access (TDMA [22]) has been developed.

When a new photometer is added to the network, a connection with the GateWay is established and a 6 s slot in the 5 min cycle is assigned. Within these 6 s, data collected by the device are sent to the GateWay, which returns a confirmation message and synchronizes the time to the next slot. The photometer will not connect again until then and enters a deep sleep state. All data collected by the devices in the network are sent to the server by the GateWay via WiFi–MQTT protocol.

Assuming a 10% error in the slot time (a total sending time of 6.6 s), each GateWay can accept up to 45 devices using the protocol developed in this work. A test network of 20 SG-WAS LoRa has been successfully established around the Teide Observatory (OT, Canary Islands, Spain).

#### 2.1.2. WiFi and LTE-M Version

The WiFi version incorporates a communication module via the MQTT protocol. The LTE-M version has a 2G multiband card and may incorporate an additional antenna. The slotted communication algorithm described above is not used with these versions because devices are directly connected to the internet. Data are stored in each device (M1, see Figure 2) and sent to the server every hour.

### 2.2. Autonomous

The measurement and communication algorithm described above has been developed to optimize the power consumption of the device. It incorporates a solar panel at the top that recharges the internal Li-Ion battery under direct sunlight (nominal 5.5 V and 35 mA with solar irradiance of 455 W/m${}^{2}$).

**Figure 3:**
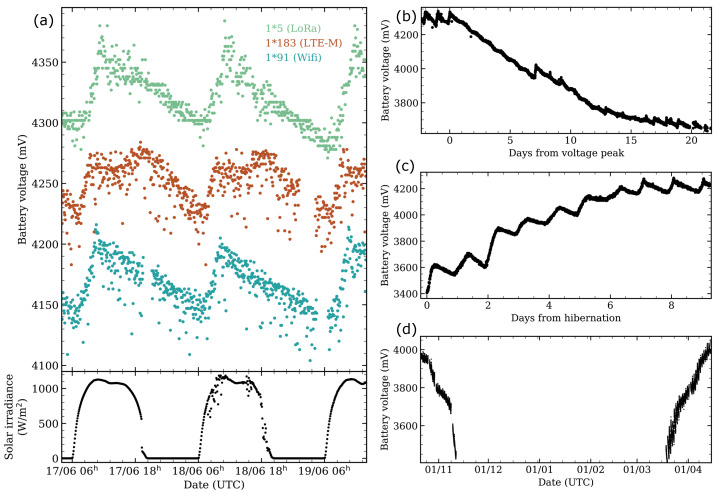
(**a**) Normal charge –discharge cycle for three photometers SG-WAS LoRa (green), LTE-M (brown), and WiFi (blue) versions located at the Teide Observatory (OT, Canary Islands, Spain). The solar irradiance, measured by the Stella telescopes weather station, is shown at the bottom; (**b**) discharge curve of an SG-WAS WiFi when not exposed to direct sunlight. It takes more than 20 days to stop taking measurements uninterruptedly until it reaches the hibernation state; (**c**) charge curve of an SG-WAS once it receives direct sunlight from the hibernation state; (**d**) voltage curve of the device 1*53 placed in the Valley of Tejeda (Gran Canaria, Spain), which is no longer exposed to direct sunlight in mid-November, remains in hibernation for 4 months and is charged in March when it begins to receive sunlight again.

Many battery life tests have been performed with the different SG-WAS versions to study their autonomy. Figure 3a shows the charge and discharge curve of three devices located at the OT, under a daytime solar irradiance ~1000 W/m${}^{2}$. In all versions, the charge lost during the night is recovered in the first 4 h of the day even in the presence of thin clouds (see a less smooth curve on 18 June), at a rate of 15–20 mV/h. Once the voltage peak is reached, the battery begins to discharge slowly at a rate of ∼2.5 mV/h. During the self-charging and discharging phases, measuring and communication is not interrupted at any time.

A complete discharge curve is shown in Figure 3b. The 1*101 (WiFi) photometer was placed inside a telescope dome that was closed during daytime. It took more than 20 days for the device to reach its battery hibernation point. This checkpoint is introduced to prevent the battery from reaching the deep discharge limit and being damaged. Once re-exposed to sunlight, the device begins to charge and send measurements again, reaching the peak voltage just a week later (Figure 3c). The hibernation phase can be maintained for months; see Figure 3d for the battery voltage of photometer 1*53 located in the Valley of Tejeda (Gran Canaria, Spain). It is located on a rocky point that does not receive sunlight during the winter and part of the autumn, so the photometer started to discharge and went into hibernation in mid-November 2020. Four months later, in mid-March 2021, it started receiving direct sunlight, sending measurements and recharging again, returning to its normal operating state. The battery stability and autonomy of the device has been extensively tested successfully.

### 2.3. Sensor

TSL237 is a silicon photodiode combined with an electrical current-to-frequency converter on a single monolithic CMOS integrated circuit. It was designed by Texas Advanced Optoelectronic Solutions [23] and is the most widely used sensor in low-cost photometers because of its low price, high sensitivity, and accuracy. TSL237 covers a spectral range between 300 nm and 1050 nm with maximum sensitivity at 700 nm, which lies between the visible and near infrared. According to the manufacturer, it can work between −25 ºC and 70 ºC, and is thermally compensated between 320 nm and 700 nm. The output signal is a square wave with a frequency that is linearly proportional to the intensity of the light (irradiance) incident on the photodiode in the range between 1 Hz and 1 MHz. A 1.8 mm diameter lens is integrated in the optical center and 0.07 mm above the photodiode.

#### 2.3.1. Linearity

According to the manufacturer [23], the sensor linearity is fulfilled in the range between 1 Hz and 1 MHz when illuminated with a spectral lamp at 524 nm at ambient temperature. This has been verified before with several SQM devices down to 19 mag/arcsec${}^{2}$ [24]. However, measuring in darker places requires studying the literality down to magnitude 22, corresponding to sub-Hz frequencies.

**Figure 4:**
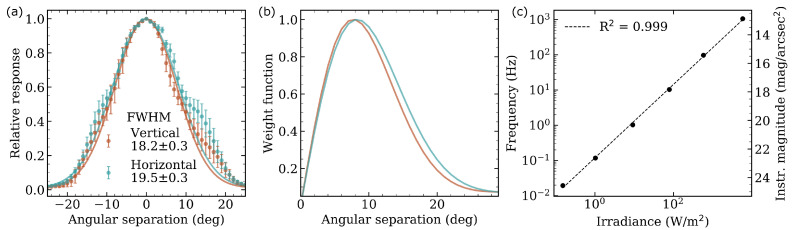
(**a**) FOV in the vertical (brown, defined in the IR window-thermometer direction) and horizontal (blue) axis, obtained in the laboratory by measuring received irradiance for different angles with respect to the optical axis. The FWHM obtained from the Gaussian fit in both axes is included. (**b**) Weight function of each FOV ring over the total flux. (**c**) Frequency measured by the TSL237 sensor against received irradiance; its approximate equivalence to magnitudes per square arcsecond is shown on the right axis. A fit is included to show the linearity of the detector to magnitudes greater than natural sky darkness.

A TSL237, an AvaSpec-ULS3648-UA-25 (AVANTES) fiber spectrometer and a HALOSTAR 50W 12V GY6.35 (OSRAM) lamp were placed inside a black box. The intensity and position of the lamp were varied to cover the range of 0.01 to 10,000 W/m${}^{2}$ received at the entry of the fiber and the sensor, which are placed together, while recording the frequency registered by the sensor. The collected measurements, including the correspondence to instrumental magnitude, are shown in Figure 4c. Linearity is maintained down to 0.01 Hz, equivalent to about 24 mag/arcsec${}^{2}$, with an $R^{2}$ very close to 1. The TSL237 sensor is therefore linear in the measurement of NSB in both light-polluted and natural dark locations.

#### 2.3.2. Angular Response

As for the SQM-L and TESS-W, a low-cost concentrator lens is located before the sensor (following the optical path), allowing more light to be collected in a narrower FOV. To verify the angular response of the device in its principal axes, it has been placed on a goniometer aligned—0${}^{\circ }$—with a point light source. Positive values indicate a clockwise rotation, being negative otherwise. Measurements have been taken from −45${}^{\circ }$ to 45${}^{\circ }$ in 1${}^{\circ }$ steps with three different devices to avoid possible random errors in the mounting of one of them. The mean values and their deviation are shown in Figure 4a. The FOV is approximately Gaussian in both axes, with a full width at half maximum (FWHM) of 18.2±0.3 deg in the vertical and 19.5±0.3 deg in the horizontal. The bumps that stand out from the fit are due to stray light reflections at very specific positions in the experimental set-up. This FWHM is close to that of SQM-L (20${}^{\circ }$ [12]) and TESS-W (17${}^{\circ }$ [13]).

Knowing the FOV, the contribution of each integrated ring, defined from the angular separation with respect to the optical axis, to the total flux measured by the device can be obtained. This is a weight function that should be considered when calculating the average radiance at the entrance of the detector plane—see, for example, Equation (7). The result on both axes is shown in Figure 4b. Although the detector response decreases with increasing angular separation, the integrated sky area is larger and the maximum is located approximately 8–9º from the optical axis. This is particularly meaningful in the presence of gradients produced by extended artificial (ALAN) or natural (twilight, moonlight, galactic plane, etc.) illumination, but less for point sources such as stars, whose fluxes are added to the rest of the sky in the integrated ring.

#### 2.3.3. Transmittance

A window and optical filter were placed in the entrance plane in the early versions of SG-WAS. This is a similar configuration to that of the SQM, with a Hoya CM-500, and TESS-W, with a dichroic filter. The empty gap between the two surfaces is exposed to condensation from any moisture that may remain inside the case. Manufacturers of the TESS-W included a heater to keep the internal temperature above 10 °C and avoid such condensation. Given the autonomy requirements of the SG-WAS, this energy-intensive solution (more power-consuming than the measurement and communication processes) is not feasible. The dichroic+window assembly has been replaced, then, by a single dichroic, which limits the spectral response of the detector and ensures proper sealing of the device.

**Figure 5:**
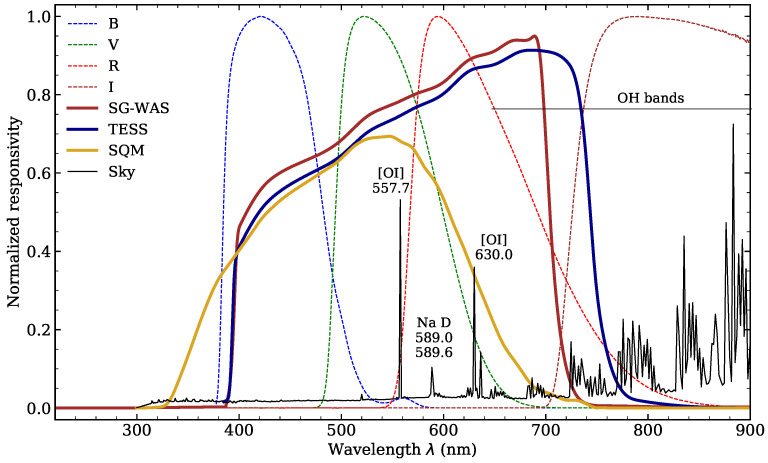
Spectral response curve of the SG-WAS. The sensitivity of the TSL237 sensor is limited to the visible range by a dichroic filter, and the window has been removed to improve the transmittance of the optical system. The transmittance of the TESS-W and SQM-L photometers and Johnson–Cousins [25] *BVRI* filters has been included as a reference, as well as the night sky spectrum obtained using the SkyCalc tool [26], where the brightest airglow lines are labeled. Adapted with permission from [10] ©AAS 2021.

This is also an opportunity to optimize the transmittance of the device, a critical consideration if an additional color filter is added to the SG-WAS optics (under development). The window causes an approximately 8% loss in transmittance and the filter, 6%. If they were considered as two independent elements, the total transmittance loss would be approximately 13%. Removing the window causes the optical system to become more sensitive, as shown in Figure 5. The spectral range of SG-WAS is fitted with *BVR* filters, from 400 to 720 nm. These filters cover the main optical airglow lines.

## 3. Results and Discussion

### 3.1. SG-WAS Astronomical Magnitudes and Uncertainties

Following standards in astronomy, brightness is usually expressed in units of magnitude per square second as follows:

(1) $$m=ZP-2.5\log_{10}\left(f-f^{D}\right),$$

where ZP is the laboratory-defined zero point of the photometer using a reference light source, and *f* and $f^{D}$ are the signal and dark frequencies of the sensor, respectively, both measured in Hz. To avoid mistakes, it is more correct to consider the SQM, TESS, and SG-WAS responses as different photometric systems. The conversion factors between photometric systems can be obtained based on the kind of spectra of the observed object [14]. Hereafter, the brightness in mag/arcsec${}^{2}$ measured in the SG-WAS passbands will be called m${}_{\mathrm{SG}}$ and Equation (1) is transformed into

(2) $$m_{\mathrm{SG}}=ZP-2.5\log_{10}\left(f_{\mathrm{SG}}-f_{\mathrm{SG}}^{D}\right).$$

At typical nighttime low operating temperatures (<20${}^{\circ }$), the value of the dark frequency, $f_{\mathrm{SG}}^{D}$, turns out to be negligible in most cases; therefore, the total error of the measurements of the SG-WAS photometer for output frequency $f_{\mathrm{SG}}$, $\delta^{2}m_{\mathrm{SG}}$, is obtained and converted to device-specific brightness units by propagating the expression (2) as follows:

(3) $$\delta^{2}m_{\mathrm{SG}}=\delta_{ZP}^{2}+\;{\left(\frac{2.5\log_{10}\left(e\right)}{f_{\mathrm{SG}}}\;\delta_{f_{\mathrm{SG}}}\right)}^{2},$$

where

(4) $$\delta_{ZP}=\mathrm{Calibration}\;\mathrm{Error}$$

(5) $$\delta m_{\mathrm{SG}}^{ins}=\;\frac{2.5\log_{10}\left(e\right)}{f_{\mathrm{SG}}}\;\delta_{f_{\mathrm{SG}}}=\mathrm{Instrumental}\;\mathrm{Error}.$$

both calibration and instrumental errors must be taken into account when determining the total uncertainty of the measurements.

### 3.2. Sky Integrating Sphere (SIS) Calibration Method

The determination of the ZP is the key issue in the reproducibility of measurements and consistency between different devices. Starting from the expression (2), and considering that the measurement of the reference device $m_{{\mathrm{SG}}_{\mathrm{ref}}}$ is expressed in the photometric absolute (AB) magnitude system, the difference between this and a simultaneous measurement of another device will be given by

(6) $$m_{\mathrm{SG}}-m_{{\mathrm{SG}}_{\mathrm{ref}}}=ZP-2.5\log_{10}\left(\frac{f_{\mathrm{SG}}-f_{\mathrm{SG}}^{D}}{f_{{\mathrm{SG}}_{\mathrm{ref}}}-f_{{\mathrm{SG}}_{\mathrm{ref}}}^{D}}\right)$$

In the comparison calibration process, we are interested in establishing the ZP value from the magnitude difference. Under the same illumination conditions, two correctly calibrated photometers, i.e., with the ZP well determined, should measure the same magnitude within the uncertainty range, so the ratio between $\left(f_{\mathrm{SG}}-f_{\mathrm{SG}}^{D}\right)/\left(f_{{\mathrm{SG}}_{\mathrm{ref}}}-f_{{\mathrm{SG}}_{\mathrm{ref}}}^{D}\right)$ is constant. The relationship between the frequency measured by the TSL237 sensor and the spectrally weighted and FOV-averaged radiance *L* at the entrance plane of the detector is given by

(7) $$f=K\int_{0}^{\infty }T\left(\lambda \right)\left[\int_{\Omega }P\left(\omega \right)L_{\lambda }\left(\omega \right)d^{2}\omega \right]d\lambda +f^{D}$$

where $L_{\lambda }\left(\omega \right)$ is the spectral radiance of the incident light field along the direction specified by the angular vector ω, P(ω) is the weight function describing the FOV of the device, normalized such that $\int_{\Omega }P\left(\omega \right)d^{2}\omega =1$, T(λ) is the spectral response of the device and *K* a constant that provides the absolute link between the converter output frequency and incident radiance [14]. Under angularly uniform radiance illumination (i):

(8) $$f=K\int_{0}^{\infty }T\left(\lambda \right)L_{\lambda }d\lambda +f^{D}$$

which is device-independent only if (ii) $f^{D}$, which varies with temperature, is the same, i.e., $f_{\mathrm{SG}}^{D}=f_{{\mathrm{SG}}_{\mathrm{ref}}}^{D}$, or negligible; (iii) there is no significant change for both devices in the spectral response T(λ), bearing in mind that the incident radiance has the same spectral distribution, i.e., $L_{\lambda }^{\mathrm{SG}}=L_{\lambda }^{{\mathrm{SG}}_{\mathrm{ref}}}$. These three conditions must be fulfilled to obtain the ZP from the difference of simultaneous measurements of two photometers.

A well-established procedure is to use a stable light source to illuminate a homogeneous surface and take simultaneous measurements between a reference photometer and the one to be calibrated, thereby establishing the *ZP* from the differences. In both the SQM-L and TESS-W calibrations, an integrating sphere is used to ensure homogeneous irradiance. In the first case, a broadband spectral lamp is used, with increasing irradiance in the wavelength range of 350 to 500 nm that remains almost constant thereafter [27] and, in the second one, a LED with a spectral response centered at 596 nm and an FWHM of 14 nm [28]. This system presents several difficulties in ensuring reproducibility and usually requires a 1-by-1 calibration.

On the one hand, a dependence on the experimental setup is introduced that may lead to increased measurement uncertainty (as reported by [24]) due, for example, to imprecise positioning or poor screening of the background stray light breaking the angular uniformity (i). Moreover, it requires careful control of the temperature at which the measurements are taken, as this may not only affect the emission spectrum of the lamp but also the dark frequency, which may not be negligible at working temperature (ii).

On the other hand, it is known that the spectral response of different photometers is not the same, so condition (iii) is not always satisfied. In the extreme case, it is not correct to obtain the *ZP* by comparing simultaneous measurements of a TESS-W and an SQM-L because their spectral response is very different and hence also the frequency measured, even under the same illumination conditions. This effect also occurs, although to a lesser extent, in photometers of the same type. When calibrating with a narrow source, as in the case of the TESS-W, it is assumed that the difference in magnitudes obtained in the spectral range of the lamp can be extrapolated to the full spectral response of the device, thus introducing an undesired and unknown systematic error. To reduce this systematic error in night sky brightness measurements, a light source with a spectral distribution similar to that of the night sky may be much more appropriate.

In this work, we have developed a novel calibration method based on simultaneous measurements of the night sky that can be used in any type of NSB device. The night sky is likely to behave as an integrating sphere if there is no light gradient in the photometer FOV. Therefore, it is necessary to avoid localized sources of both artificial (ALAN) and natural light emission (astronomical components such as the Moon or the Galaxy). All SG-WAS photometers are calibrated on zenith observations taken from the OT, where light pollution is minimal [10] and condition (i) satisfied (further details on the calibration procedure are omitted because of a patent-pending process). When taking simultaneous nighttime measurements, not only is the temperature the same in all devices, but also, at an altitude of 2500 m, it does not get high enough for the dark frequency to be significant. In addition, many photometers (currently up to 100 devices at the OT) can be calibrated at the same time as there is space available on the optical table.

The most meaningful point of this method is that the calibration measurements are taken with the same spectrum of the night sky. This allows the systematic error to be very well constrained to the absolute calibration of the reference device. The difference between the measurements is thought to come mainly from the small variations in the spectral response of the photometer, resulting in a variation of the magnitude value with the changes that take place in the night sky spectrum over time.

### 3.3. SG-WAS Calibration Error

**Figure 6:**
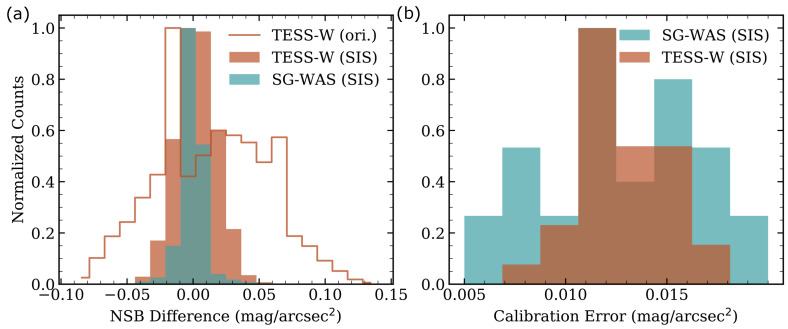
(**a**) Distribution of differences in simultaneous NSB measurements between pairs of TESS-W (brown) and SG-WAS (blue) photometers after calibrating them using the Sky Integrating Sphere (SIS) method. The distribution of TESS-W photometer pairs with the original calibration (ori.) given by the manufacturer is also shown unfilled. (**b**) Distribution of calibration errors for both photometers obtained with the SIS method.

The SIS method was used to obtain the *ZP* of 10 TESS-W and 21 SG-WAS photometers. The differences between simultaneous zenith measurements taken between pairs of photometers under dark conditions (following the definition described in [10]) for the period 18 January–26 May 2021 and for TESS-W and between 14 April–10 June 2021 for SG-WAS are shown in Figure 6a. Both distributions are centered at 0, as expected, and have a standard deviation of 0.01 mag/arcsec${}^{2}$ in the case of SG-WAS and 0.02 mag/arcsec${}^{2}$ for TESS-W. These values are greater than the combination of instrumental errors (more detail in the next section) and are due to the effect of variations in the night sky spectrum captured by a slightly different spectral response in the devices. The uncertainty in the determination of the *ZP* by the SIS method does not exceed 0.02 mag/arcsec${}^{2}$, as it is shown in Figure 6b. This is a reduction of more than a factor 2 of the 0.044 mag/arcsec${}^{2}$ reported as the calibration error in the TESS-W [14,28] and a factor of 5 of the SQM [24].

The original calibration of the TESS-W, which is made by the manufacturer in the Laboratory for Scientific Advanced Instrumentation (Laboratorio de Instrumentación Científica Avanzada, LICA) of Universidad Complutense de Madrid (UCM, Spain), presents a more scattered distribution in the differences, with a standard deviation of 0.042 mag/arcsec${}^{2}$ (see the unfilled distribution in Figure 6).

**Figure 7:**
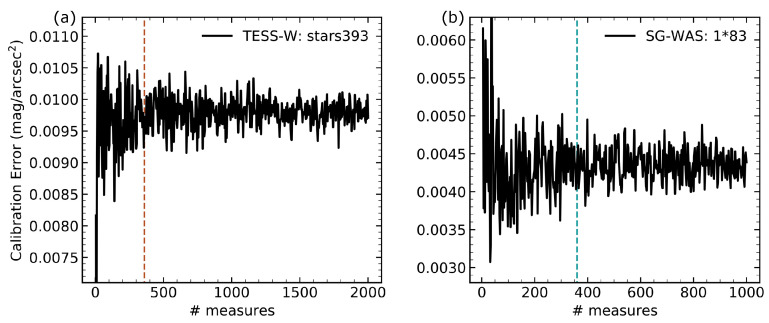
Calibration error obtained with the SIS method for a TESS-W photometer (**a**) and an SG-WAS (**b**), as a function of the number of measurements taken randomly. It is considered that the lower limit is reached from 360 onward (dashed vertical lines), corresponding to 6 and 30 h of night sky measurements, respectively.

Figure 7 shows the variation of the calibration error for a TESS-W and an SG-WAS as a function of the number of measurements considered, taken randomly from the whole set. The choice of these particular devices is arbitrary, and their reproducibility has been checked for all other available photometers. From 360 measurements onward, the *ZP* determination is considered to have reached its accuracy limit and the calibration error cannot be further reduced. Given the temporal resolution of the photometers, this is equivalent to 6 h of measurements in the TESS-W and 30 h in the SG-WAS. Although the time available to take such measurements depends on different astronomical and atmospheric components, it has been verified that this interval is easily achievable in one week at the OT, regardless of the time of year. The SIS method presented in this work not only reduce the calibration uncertainty but also allows a massive calibration to be performed –currently up to 100 devices– by taking simultaneous measurements for no longer than one clear week.

### 3.4. SG-WAS Instrumental Error

**Figure 8:**
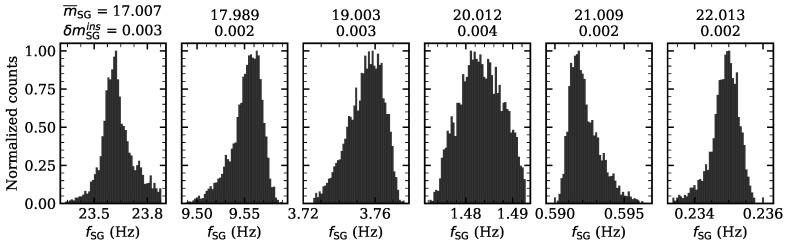
Distribution of 5000 laboratory frequency measurements with light intensities for $m_{\mathrm{SG}}$ values included between 17 and 22 mag/arcsec${}^{2}$ range in steps of 1 mag/arcsec${}^{2}$. The standard deviation in magnitude units, corresponding to the instrumental uncertainty, is included on top.

A statistical study of the data has been carried out to characterize the instrumental error of the system (sensor+optics). For this purpose, 5000 laboratory frequency measurements were taken for magnitude values between 17 and 22 mag/arcsec${}^{2}$ (as expected in the field concerned) in steps of 1 mag/arcsec${}^{2}$. The optical setup is similar to that used in previous sections. For each set of 5000 measurements, a histogram has been obtained and represented in Figure 8. Statistical parameters are shown in Table 1.

The calculated standard deviation values for each distribution magnitude is lower than 0.004 mag/arcsec${}^{2}$ (see Table 1). This experiment therefore shows that the instrumental error of the SG-WAS photometer is lower than a few milli-magnitudes. Taking into account that this laboratory error calculation can still be affected by other factors such as the power supply error, the stability of the lamp used, the design of the experiment (background stray light), electromagnetic interference, etc., it should be considered an upper limit to the instrumental error.

The new measurement method developed for the SG-WAS allows us to obtain the average of ten continuous measurements of NSB and also its standard deviation. The time taken by the device to take a measurement is inversely proportional to the frequency. Following Table 1, it takes approximately 4.5 s to take one measurement at magnitude 22, 1.7 s at magnitude 21, and so on. In total, ten continuous measurements at magnitude 22 are taken in about 45 s. As the possible variations in sky brightness produced by airglow are not measurable on this time scale [10], the standard deviation of these measurements corresponds only to the instrumental error, which is provided along with the NSB every 5 min in the SG-WAS.

### 3.5. Night Sky Brightness Measurements

**Figure 9:**
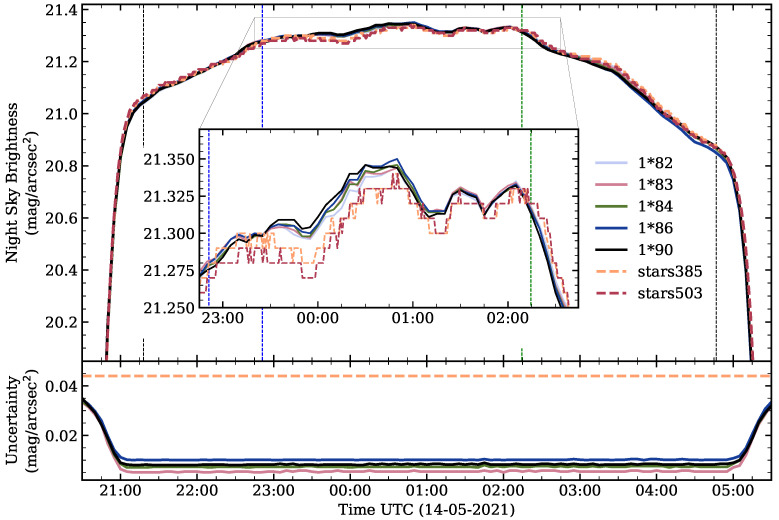
NSB curve of five SG-WAS (1*X) and two TESS-W (starsX) photometers taken on 2021 May 14 from Teide Observatory (Canary Islands, Spain). The vertical lines indicate the end (**left**) and beginning (**right**) of astronomical twilight (black), the leaving of the zodiacal light (blue) and the entrance of the Galaxy (green) in the FOV of the device. The inner graph shows the dark period [10] enlarged. The SG-WAS curves are smoother than those of TESS-W as a consequence of the 10-measurement averaging process. The lower plot shows the total uncertainty in the measurements, including the instrumental error—calculated for each SG-WAS measurement and its upper bound of 0.004 mag/arcsec${}^{2}$ for TESS-W—and the calibration error—obtained by the SIS method for SG-WAS and 0.044 mag/arcsec${}^{2}$ for TESS-W.

More than one-hundred SG-WAS photometers were installed at the OT during 2021 to test their resistance to extreme weather stress conditions (snow, ice, strong wind, high solar radiation, among others) and the reliability of their NSB measurements. Figure 9 shows the zenithal brightness curve on 14 May 2021 for five SG-WAS and two TESS-W placed together. In the inner plot, the dark and clear period (as defined by [10]) is shown enlarged. Note that the SG-WAS curves are smoother than those of TESS-W, which is a result of the averaging process. Variations associated with airglow are clearly visible in both devices, although some differences between them are appreciable. This is a consequence of the slight difference in the spectral response of both devices. The SG-WAS curves are very similar to each other, with their difference being less than one-hundredth of a magnitude.

The lower plot shows the total uncertainty in the measurements, including the instrumental error (calculated for each SG-WAS measurement and its upper bound of 0.004 mag/arcsec${}^{2}$ for TESS-W) and the calibration error (obtained by the SIS method for SG-WAS and 0.044 mag/arcsec${}^{2}$ for TESS-W). The uncertainties of the SG-WAS measurements are below 0.02 throughout the night, except at twilight, when the rapid change in sky brightness increases the standard deviation of the ten measurements. These curves have been found to be very nearly the same for all other SG-WAS photometers, regardless of version and series.

### 3.6. Light Pollution Laboratory: IoT-EELab

The measurements collected by the EELabs and STARS4ALL networks are openly available in real-time at the IoT-EELab website (data.eelabs.eu, accessed on 28 June 2021). It is an interactive dashboard that collects, controls, and analyses data from hundreds of internet-connected NSB sensors and several all-sky cameras. There are more than 170 million entries available containing brightness data collected for more than 500 photometers (400 TESS and 100 SG-WAS).

IoT-EELab includes a simple reading page with NSB data from a single location/single date with calculation of the lunar phase, mean and median values of NSB during the astronomical night, slopes of brightness curves in different time intervals, sunset and sunrise hours, and percentage of cloud cover. Furthermore, there are statistical tools to obtain histograms, “hourglass” diagrams, “jellyfish” plots, and monthly and annual NSB trends, among other features. All raw and filtered data can be downloaded in standard format. IoT-EELab provides a unique opportunity for researchers to experiment with new and existing light pollution datasets.

## 4. Conclusions

In this work, the characterization of the new SG-WAS, a low-cost NSB photometer based on the TSL237 sensor (similar to SQM and TESS), has been carried out. The main conclusions are summarized as follows:

- SG-WAS is the first wireless NSB sensor to communicate via LoRa, WiFI, or LTE-M and be powered by solar energy. A measurement every 5 min is taken continuously and sent using a Time Division Multiple Access algorithm to avoid packet collision.
- The device recharges to peak voltage in just 4 h with direct solar irradiance after a full night of operation. It can stay up to 20 days in darkness while taking measurements, subsequently remain in hibernation for months before returning to operation once it is illuminated again.
- Its optical design is very similar to the TESS-W, with a FOV approximately 19${}^{\circ }$ (FWHM) and a slightly less red spectral range, from 400 to 720 nm. The window has been removed to prevent condensation on the dichroic and increase transmittance.
- A new Sky Integrating Sphere (SIS) calibration method (patent-pending) has been designed and demonstrated to achieve calibration errors in TESS-W and SG-WAS below 0.02 mag/arcsec${}^{2}$.
- The robustness to adverse weather conditions and the stability of its measurements have been demonstrated in field tests. Taking the average and uncertainty of ten continuous measurements makes the NSB curves smoother and avoids spikes. Differences between simultaneous measurements of several SG-WAS photometers have been found to have a standard deviation of 0.01 mag/arcsec${}^{2}$, several times smaller than their predecessors.

## Figures and Tables

**Table 1:** Frequency standard deviation values for each distribution. Following Equation (5), the SG-WAS instrumental magnitude, $\delta m_{\mathrm{SG}}^{ins}$, was also calculated.

Frecuency (Hz)	Magnitude (mag/arcsec${}^{2}$)	$\delta_{f_{\mathrm{SG}}}=\sigma_{f_{\mathrm{SG}}}$ (Hz)	$\delta m_{\mathrm{SG}}^{\mathrm{ins}}$ (mag/arcsec${}^{2}$)
9.5563	17.989	0.0134	0.002
3.7564	19.003	0.0100	0.003
1.4833	20.012	0.0053	0.004
0.5918	21.009	0.0009	0.002
0.2350	22.013	0.0004	0.002

## Data Availability

Access and download of the data used is openly available at data.eelabs.eu, accessed on 18 August 2021.
